# Linoleic Acid-Induced Ultra-Weak Photon Emission from *Chlamydomonas reinhardtii* as a Tool for Monitoring of Lipid Peroxidation in the Cell Membranes

**DOI:** 10.1371/journal.pone.0022345

**Published:** 2011-07-25

**Authors:** Ankush Prasad, Pavel Pospíšil

**Affiliations:** Department of Biophysics, Centre of the Region Haná for Biotechnological and Agricultural Research, Faculty of Science, Palacký University, Olomouc, Czech Republic; University of Geneva, Switzerland

## Abstract

Reactive oxygen species formed as a response to various abiotic and biotic stresses cause an oxidative damage of cellular component such are lipids, proteins and nucleic acids. Lipid peroxidation is considered as one of the major processes responsible for the oxidative damage of the polyunsaturated fatty acid in the cell membranes. Various methods such as a loss of polyunsaturated fatty acids, amount of the primary and the secondary products are used to monitor the level of lipid peroxidation. To investigate the use of ultra-weak photon emission as a non-invasive tool for monitoring of lipid peroxidation, the involvement of lipid peroxidation in ultra-weak photon emission was studied in the unicellular green alga *Chlamydomonas reinhardtii*. Lipid peroxidation initiated by addition of exogenous linoleic acid to the cells was monitored by ultra-weak photon emission measured with the employment of highly sensitive charged couple device camera and photomultiplier tube. It was found that the addition of linoleic acid to the cells significantly increased the ultra-weak photon emission that correlates with the accumulation of lipid peroxidation product as measured using thiobarbituric acid assay. Scavenging of hydroxyl radical by mannitol, inhibition of intrinsic lipoxygenase by catechol and removal of molecular oxygen considerably suppressed ultra-weak photon emission measured after the addition of linoleic acid. The photon emission dominated at the red region of the spectrum with emission maximum at 680 nm. These observations reveal that the oxidation of linoleic acid by hydroxyl radical and intrinsic lipoxygenase results in the ultra-weak photon emission. Electronically excited species such as excited triplet carbonyls are the likely candidates for the primary excited species formed during the lipid peroxidation, whereas chlorophylls are the final emitters of photons. We propose here that the ultra-weak photon emission can be used as a non-invasive tool for the detection of lipid peroxidation in the cell membranes.

## Introduction

The response of cyanobacteria, algae and plants to the abiotic and biotic stress environmental factors is associated with the formation of reactive oxygen species (ROS) [1], [2], [3], [4], [5]. When ROS are not properly scavenged by low molecular mass antioxidant (ascorbate, tocopherol, phenol) or antioxidant enzymes (superoxide dismutase, peroxidases, catalase), the excessive production of ROS is responsible for the oxidative damage of cellular components [6]. The main cellular components susceptible to the oxidative damage by ROS are lipids, proteins and nucleic acids [7], [8], [9], [10]. Typically, the oxidation of polyunsaturated fatty acid is initiated by radical ROS (hydroxyl radical, perhydroxyl radical), non-radical ROS (singlet oxygen, hydrogen peroxide) or by enzymatic reaction pathway (lipoxygenase) [11], [12], [13]. The non-enzymatic and enzymatic lipid peroxidation results in the formation of lipid alkyl radical (L^•^) known to form lipid peroxyl radical (LOO^•^) by interaction with molecular oxygen. The attack of lipid peroxyl radical on another polyunsaturated fatty acids results in the formation of lipid hydroperoxide [11], [14], [15], [16].

Several techniques have been developed to monitor lipid peroxidation under *in vitro* conditions. These techniques are based mainly on the detection of 1) loss of the substrate (polyunsaturated fatty acid), 2) formation of the primary peroxidation products (lipid peroxides) and 3) formation of the secondary peroxidation products (aldehyde, epoxide, keto or hydroxy compounds) [17]. In spite of the fact that the mechanistic principles of lipid peroxidation are well described under *in vitro* conditions, their application under *in vivo* conditions has raised a number of the unresolved issues [18], [19]. Under *in vivo* conditions, chemiluminescence techniques have been previously employed to study the lipid peroxidation [20], [21]. Due to the fact that chemiluminescence signal is very weak, the chemiluminescence has been denoted as ultra-weak photon emission [22], [23], [24]. As terminology for the ultra-weak photon emission is not unique so far, the other terms such as low-level biological chemiluminescence, ultra-weak bio-chemiluminescence or more widespread term biophoton emission have been frequently used [25], [26], [27], [28], [29], [30].

In the past, various types of chemical and physical probes were used to enhance the photon emission [31], [32], [33], [34]. Chemical probes are capable to enter in the lipid reaction chain and form excited state by its interaction with the product of lipid peroxidation. Luminol has been employed as the most frequently used chemical probe to enhance the chemiluminescence signal of lipid peroxidation [31]. On the opposite side, the physical probes can accept an excitation energy from the excited product of lipid peroxidation such as triplet excited carbonyl (^3^(C = O)^*^) and singlet oxygen (^1^O_2_) and emit the photons with a much higher quantum efficiency (hundred and thousand time). Among of the physical probe the fluorescence dye, coumarin was employed as an efficient enhancer of chemiluminescence signal of lipid peroxidation [32]. Alternative approach to enhance chemiluminescence signal was exposure of photosynthetic organisms to high temperature. High temperature-induced chemiluminescence has been successfully applied for the detection of lipid peroxidation [34], [35]. Two major bands in the high temperature-induced chemiluminescence has been precisely described in the temperature range of 70–90°C and 120–140°C [35], [36], [37], [38].

Recent development in the detection techniques enables to use chemiluminescence signal for the study of lipid peroxidation without the participation of exogenous enhancers [39], [40], [41], [42]. Using sensitive charged coupled device (CCD) camera, Flor-Henry and co-workers (2004) demonstrated that the mechanical wounding of *Arabidopsis thaliana* leaves caused a significant increase in the ultra-weak photon emission. The authors proposed that the products of lipid peroxidation such as ^3^(C = O)^*^ might be responsible for photon emission. Havaux (2006) demonstrated that the ultra-weak photon emission and the formation of secondary end product of lipid peroxidation malondialdehyde (MDA) from *Arabidopsis thaliana* double mutant lacking both ascorbate and zeaxanthin were significantly higher, when compared to photoresistant wild type. An alternative explanation for the ultra-weak photon emission was provided by Bennett and co-worker (2005), who related ultra-weak photon emission to the gene-for-gene mediated hypersensitive cell death.

The authors proposed that the ultra-weak photon emission is related to the formation of reactive nitrogen species (RNS); however, the participation of ROS-related lipid peroxidatiion was not completely ruled out. The hypothesis has been extended by Mansfield (2005), who made a correlation between the hypersensitive reaction leading to the generation of RNS and the lipid peroxidation leading to the ultra-weak photon emission. Using ROS scavengers, Kobayashi et al. (2007) demonstrated that ROS generated during the oxidative burst of hypersensitive reaction are involved in the ultra-weak photon emission. As far as we know, no direct evidence on the involvement of lipid peroxidation in the ultra-weak photon emission from photosynthetic organisms has been provided yet.

Here, we provide direct evidence on the involvement of lipid peroxidation in the ultra-weak photon emission from the unicellular green alga *Chlamydomonas reinhardtii*. The presented data show that the addition of linoleic acid in the cells significantly enhanced the ultra-weak photon emission. The ultra-weak photon emission was considerably suppressed by scavenging of HO^•^, inhibition of intrinsic lipoxygenase and removal of molecular oxygen. Based on the presented results it is proposed that the ultra-weak photon emission can serve as a powerful non-invasive tool for monitoring of lipid peroxidation in cyanobacteria, algae and plants.

## Results

### Spontaneous ultra-weak photon emission from intact *Chlamydomonas reinhardtii* cells

**Figure 1:**
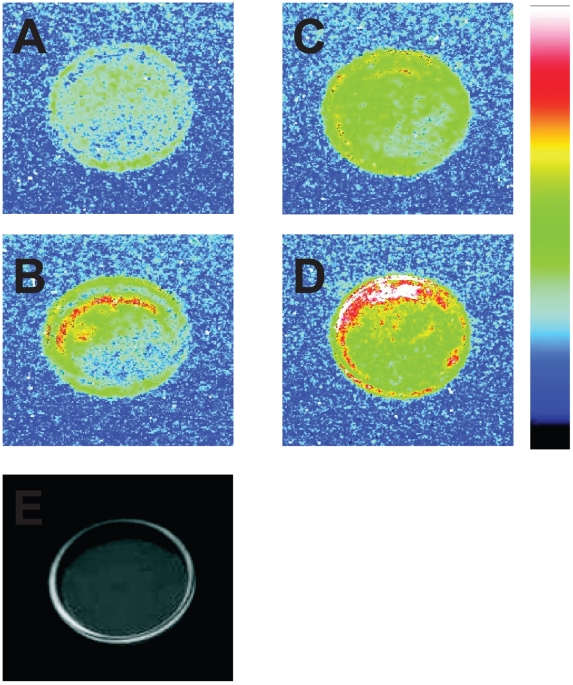
Two-dimensional imaging of the ultra-weak photon emission from the intact (A, C) and the disrupted (B, D) cells measured in the absence (A, B) and the presence (C, D) of linoleic acid. Prior to the measurements, the cells suspended in Tris-Acetate Phosphate buffer (pH 7.2) were placed on the Petri dish and kept for 30 min in the dark. In (B, D), prior to the dark adaptation the cells were frozen in liquid nitrogen and subsequently warmed to room temperature. In (C, D), 2 mM linoleic acid was added to the cells prior to the measurements. (E) represents photograph of the cells placed on the Petri dish taken under weak light illumination. Ultra-weak photon emission imaging was measured using a highly sensitive CCD camera with an integration time of 30 min.

**Figure 2:**
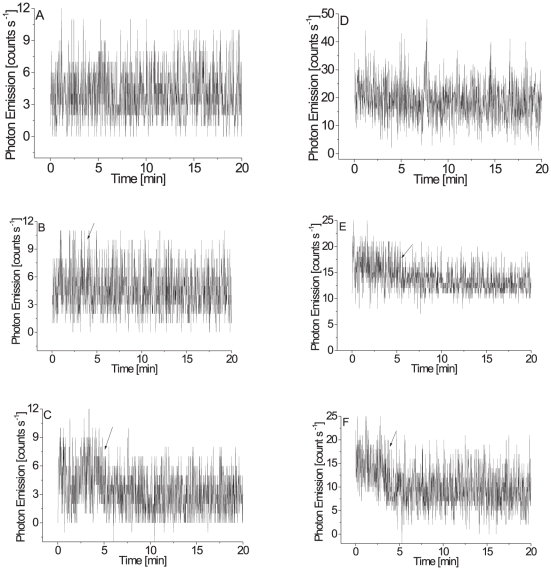
Ultra-weak photon emission from the intact (A) and the disrupted (D) cells. Prior to the measurements, the cells suspended in Tris-Acetate Phosphate buffer (pH 7.2) were placed on the Petri dish and kept for 30 min in the dark. In (D), prior to the dark adaptation the cells were frozen in liquid nitrogen and subsequently warmed to room temperature. Ultra-weak photon emission was measured using a highly sensitive PMT tube. Effect of 200 mM mannitol on the ultra-weak photon emission from the intact (B) and disrupted (C) cells. Ultra-weak photon emission under the effect of 5 mM catechol were measured from the intact (E) and disrupted (F) cells. The arrow indicates the interval at which mannitol and catechol were subsequently added.

Spontaneous ultra-weak photon emission was measured in the green alga *Chlamydomonas reinhardtii* using CCD camera. Figure 1 shows two-dimensional image of ultra-weak photon emission (A–D) and corresponding photograph (E) of the cells placed on the Petri dish. Two-dimensional image of ultra-weak photon emission indicates that the cells spontaneously emit photons (Fig. 1A). To quantify ultra-weak photon emission, the photon emission was measured using highly sensitive photomultiplier tube (PMT). When the cells were placed below the PMT window, the count rate of 4 counts s^−1^ was observed (Fig. 2A). The count rate observed from the pure growth media was 2 counts s^−1^, the value of which is comparable to the dark count (data not shown). After subtraction of the count rate from the growth media, the spontaneous ultra-weak photon emission from the cells was determined to be 2 counts s^−1^. The effect of mannitol, HO^•^ scavenger, (Fig. 2B) and catechol, an inhibitor of lipoxygenase, (Fig. 2C) on the ultra-weak photon emission was studied in the intact cells which show no significant decrease in the ultra-weak photon emission. The observation that the spontaneous ultra-weak photon emission remains unchanged during the several hours confirmed that the spontaneous ultra-weak photon emission is an intrinsic property of the cells.

### Spontaneous ultra-weak photon emission from disrupted *Clamydomonas reinhardtii* cells

To study the effect of cell disruption on the spontaneous ultra-weak photon emission, the photon emission was measured in the cells previously exposed to the mechanical disruption under liquid nitrogen. When the cells were immersed in liquid nitrogen and subsequently warmed to room temperature, the spontaneous ultra-weak photon emission was enhanced (Fig. 1B). The measurement of ultra-weak photon emission using PMT shows that after the cell disruption the count rate was 15 counts s^−1^ (Fig. 2D). These observations reveal that the cell disruption results in the enhancement in spontaneous ultra-weak photon emission. The effect of mannitol (Fig. 2E) and catechol (Fig. 2F) on the ultra-weak photon emission was studied in the disrupted cells which shows slight decrease in the ultra-weak photon emission. However, to observe a significant difference in the ultra-weak photon emission in the presence of mannitol and catechol, linoleic acid-induced ultra-weak photon emission was employed.

### Effect of linoleic acid on ultra-weak photon emission from *Chlamydomonas reinhardtii* cells

**Figure 3:**
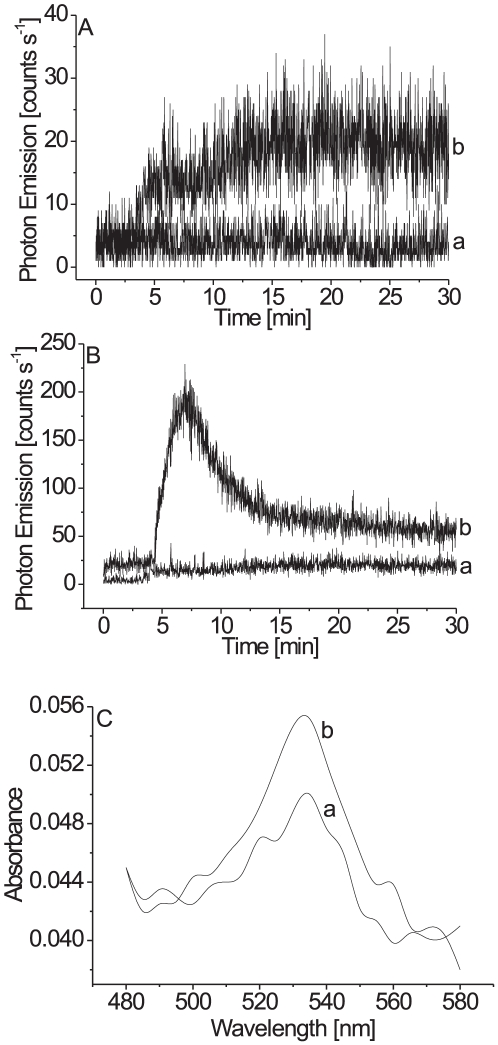
Effect of exogenous linoleic acid on the ultra-weak photon emission from the intact (A) and the disrupted cells (B). In A (trace b) and B (trace b), 2 mM linoleic acid was added to the cells prior to the measurements. Other experimental conditions as in Fig. 1. (C), Absorption difference spectra of the (TBA)_2_-MDA adduct measured in the cells in the intact (trace a) and the disrupted cells (trace b). Absorption difference spectrum represents the difference in the absorption spectrum of the (TBA)_2_-MDA adduct obtained before and after the addition of linoleic acid.

To test the involvement of lipid peroxidation in the ultra-weak photon emission, the effect of exogenous linoleic acid on the ultra-weak photon emission was studied in the both intact and disrupted cells. When linoleic acid was added to the intact cells, an enhancement in the two-dimensional ultra-weak photon emission was observed (Fig. 1C). Figure 3A (trace b) shows that the addition of linoleic acid to the intact cells results in a gradual increase in photon emission to 15 counts s^−1^. Similarly, the addition of linoleic acid to the disrupted cells caused a significant increase in the two-dimensional ultra-weak photon emission (Fig. 1D). Figure 3B (trace b) shows that the addition of linoleic acid to the disrupted cells results in the enhancement in photon emission to 220 counts s^−1^ followed by a gradual decrease. Based on these observations it is concluded that the oxidation of linoleic acid results in the enhancement in ultra-weak photon emission with the most pronounced effect observed in the disrupted cells.

### Determination of (TBA)_2_-MDA adduct in *Chlamydomonas reinhardtii* cells

Spectroscopic detection of the thiobarbituric acid reactive substances (TBARS) was used to monitor the formation of lipid hydroperoxides in the both intact and disrupted cells. In this method, the decomposition of lipid peroxides results in the formation of MDA, which reacts with thiobarbituric acid forming (TBA)_2_-MDA adduct [11], [43]. Figure 3C shows the absorption spectrum of (TBA)_2_-MDA adduct obtained in the intact cells (trace a) and the disrupted cells (trace b) after the addition of linoleic acid with the unique absorption maximum at 532 nm. The absorption spectra show that the enhancement in absorbance at 532 nm observed in the disrupted cells was considerably higher, when compared to the intact cells. The quantitative determination of (TBA)_2_-MDA adduct shows that the addition of linoleic acid to the intact cells results in the formation of 40±2 nmol MDA/2.1×10^8^ cells, whereas it was increased to 100±10 nmol MDA/2.1×10^8^ cells in the disrupted cells. These results indicate that the oxidation of linoleic acid by intrinsic lipoxygenase initiates lipid peroxidation in the cells.

### Effect of mannitol, lipoxygenase inhibitor and molecular oxygen on ultra-weak photon emission from *Chlamydomonas reinhardtii* cells

**Figure 4:**
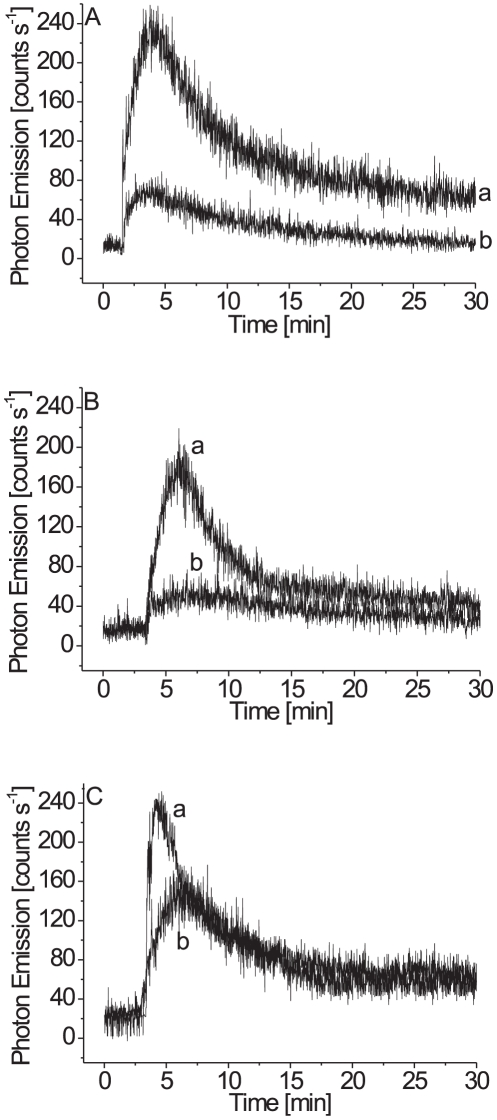
Effect of mannitol (A), catechol (B) and molecular oxygen (C) on the ultra-weak photon emission from the disrupted cells measured in the presence of linoleic acid. In (A), ultra-weak photon emission was observed in the absence (trace a) and the presence of 200 mM mannitol (trace b). In (B), ultra-weak photon emission was observed in the absence (trace a) and the presence (trace b) of 5 mM catechol. Catechol was added to the cells prior to the addition of linoleic acid. Other experimental conditions as in Fig. 1. In (C), ultra-weak photon emission was observed under aerobic (trace a) and anaerobic (trace b) conditions. To remove molecular oxygen, the cells were treated with 1 mM glucose and 50 U ml^−1^ glucose oxidase for 15 min. Other experimental conditions as in Fig. 1.

To study the involvement of hydroxyl radical (HO^•^) in the ultra-weak photon emission, the effect of mannitol, HO^•^ scavenger, on the ultra-weak photon emission was studied in the disrupted cells. The scavenging of HO^•^ by addition of mannitol in the disrupted cells caused a pronounced suppression in ultra-weak photon emission (Fig. 4A, trace b). These observations indicate that HO^•^ is involved in the ultra-weak photon emission.

The involvement of intrinsic lipoxygenase in the ultra-weak photon emission was explored by monitoring the effect of catechol, an inhibitor of lipoxygenase, on the ultra-weak photon emission. The addition of catechol to the disrupted cells results in the significant suppression in the ultra-weak photon emission (Fig. 4B, trace b). These observations reveal that the oxidation of linoleic acid by intrinsic lipoxygenase is involved in the ultra-weak photon emission.

To test the involvement of molecular oxygen in the ultra-weak photon emission, the photon emission was measured after removal of molecular oxygen using enzyme system glucose/glucose oxidase. When the disrupted cells were incubated with glucose/glucose oxidase for 15 min, a pronounced decline in the ultra-weak photon emission was observed (Fig. 4C, trace b). This observation shows that molecular oxygen participate in the ultra-weak photon emission.

### Spectral properties and effect of histidine on ultra-weak photon emission from *Chlamydomonas reinhardtii* cells

**Figure 5:**
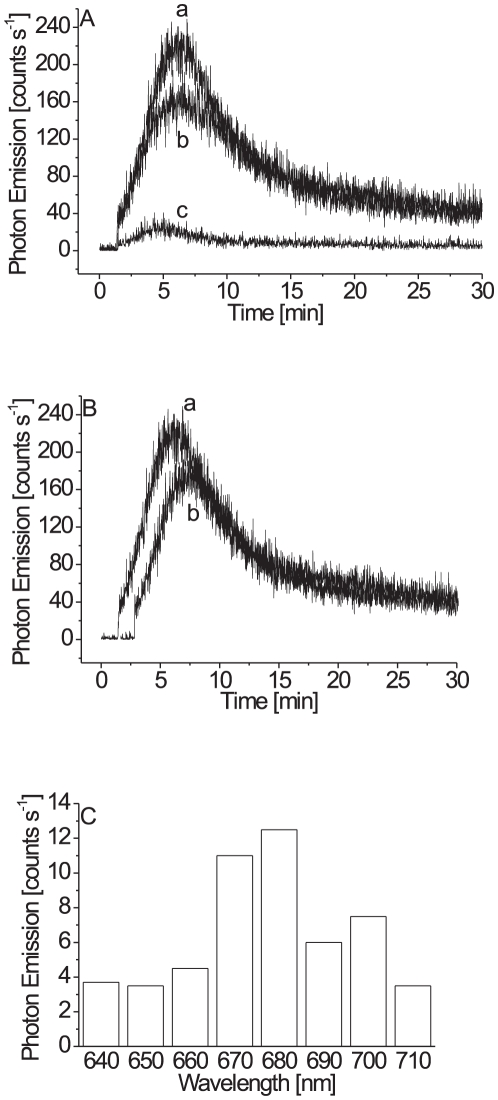
Spectral properties (A,C) and the effect of histidine (B) on the ultra-weak photon emission from the disrupted cells measured in the presence of linoleic acid. In (A), ultra-weak photon emission was measured in the absence of filter (trace a) and in the presence of filters passing the photon emission in the range of wavelength >600 nm (trace b) and 310–600 nm (trace c). Other experimental conditions as in Fig. 1. In (B), ultra-weak photon emission was observed in the absence (trace a) and presence of 10 mM histidine (trace b). In (C), ultra-weak photon emission was measured in the red region of the spectrum from 640 nm to 710 nm using a set of interference filters with a bandwidth of 10 nm. The different bars on the bar graph represent the photon emission observed with the interference filters centered at 641 nm, 651 nm, 662 nm, 671 nm, 683 nm, 691 nm and 702 nm.

To characterize excited species involved in the ultra-weak photon emission, the spectral properties of ultra-weak photon emission were measured in the disrupted cells. To distinguish between the photon emission from the blue and the red region of the spectrum, the cut-off filters were mounted in the front of PMT. When the blue region of the spectrum was cut off, no significant change in the ultra-weak photon emission was observed (Fig. 5A, trace b). Interestingly, when the red region of the spectrum was cut off, the ultra-weak photon emission was significantly suppressed (Fig. 5A, trace c). These observations indicate that the excited species with the photon emission in the red region of the spectrum contribute to the ultra-weak photon emission.

To explore the involvement of ^1^O_2_ in the ultra-weak photon emission, the effect of histidine, ^1^O_2_ scavenger, on the ultra-weak photon emission was measured in the disrupted cells. When histidine was added to the disrupted cells, no change in the ultra-weak photon emission was observed (Fig. 5B, trace b). These observations show that ^1^O_2_ is unlikely involved in the ultra-weak photon emission.

To investigate the photon emission in the red region of the spectrum in more detail, the set of interference filters in the range of 640 to 710 nm was used. Figure 5C shows that the maximum photon emission peak at the red region of the spectrum is at 680 nm. Based on this observation, it is assumed that emission originates from chlorophyll molecules.

## Discussion

Lipid peroxidation is considered as a free radical chain reaction initiated by the oxidation of polyunsaturated fatty acid [11], [15], [16]. Linoleic acid is one of the main fatty acids in the membrane, which has unsaturated bonds between C9–C10 and C12–C13 carbons. The oxidation of linoleic acid followed by the hydrogen abstraction results in the formation of L^•^, which in the presence of molecular oxygen forms LOO^•^. The oxidation of linoleic acid proceeds via the non-enzymatic reaction pathway mediated by free oxygen radicals or via the enzymatic reaction pathway mediated by lipoxygenase.

### Non-enzymatic oxidation of linoleic acid

**Figure 6:**
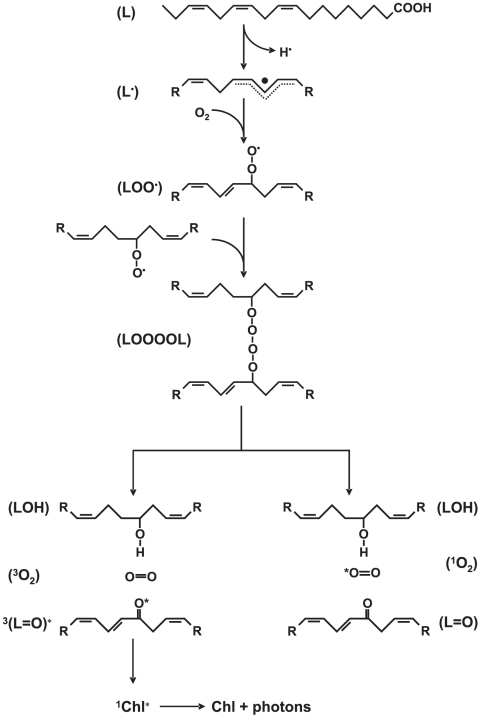
Mechanism of generation of electronically excited species by oxidation of polyunsaturated fatty acids. Abstraction of allylic hydrogen from polyunsaturated fatty acids (L) results in the formation of lipid alkyl radical (L^•^). Lipid alkyl radical reacts with molecular oxygen to generate a lipid peroxyl radical (LOO^•^). Peroxyl radical reacts with the adjacent peroxyl radical by the Russell-type mechanism forming carbonyl, molecular oxygen and lipid hydroxides (LOH). The carbonyls are formed either in the triplet excited (L = O^*^) or the ground (L = O) state, whereas molecular oxygen is correspondingly in the triplet ground state (^3^O_2_) or the singlet excited state (^1^O_2_). The excitation energy transfer from the triplet excited carbonyls to chlorophyll molecules results in the formation of singlet excited state of chlorophyll (^1^Chl^*^). An electronic transitions from the singlet excited state to the ground state of chlorophyll is accompanied by the emission of photons in the red region of the spectrum.

In the non-enzymatic reaction pathway, the free oxygen radical readily abstracts a hydrogen atom from the hydrocarbon chain of polyunsaturated fatty acids, leading to the formation of L^•^ (Fig. 6). Our observation that the scavenger of HO^•^ (mannitol) significantly suppressed the ultra-weak photon emission indicates that the lipid peroxidation is initiated by HO^•^ (Fig. 4A, trace b). Hydroxyl radical is formed by the reduction of H_2_O_2_ by free metals through Fenton-type chemistry [44]. Lipid alkyl radical reacts with molecular oxygen at the diffusion-limited rate to form LOO^•^ (Fig. 6). The observation that the removal of molecular oxygen results in the significant suppression in ultra-weak photon emission indicates that molecular oxygen is involved in the ultra-weak photon emission (Fig. 4C, trace b).

### Enzymatic oxidation of linoleic acid

In the enzymatic reaction pathway, the oxidation of polyunsaturated fatty acids is catalyzed by lipoxygenase. The first step in the enzymatic reaction is the abstraction of the hydrogen atom from C-11 by the ferric non-heme iron of the enzyme (Fe^3+^–OH) to generate L^•^, whereas the active site of the enzyme is reduced to the ferrous non-heme iron (Fe^2+^–OH_2_) [45]. Catechol (an inhibitor of lipoxygenase) is known to bind to the ferric non-heme iron of the enzyme and thus prevents the formation of L^•^ [46]. Our observation that catechol considerably suppressed the ultra-weak photon emission indicates that the formation of L^•^ is catalyzed by the intrinsic lipoxygenase (Fig. 4B, trace b). The interaction of L^•^ with molecular oxygen results in the formation of LOO^•^ and the re-oxidation of ferrous non-heme iron (Fe^2+^–OH_2_) to ferric non-heme iron (Fe^3+^–OH). The observation that the removal of molecular oxygen significantly suppressed the ultra-weak photon emission indicates that molecular oxygen is involved in the ultra-weak photon emission (Fig. 4C, trace b).

### Ultra-weak photon emission reflects formation of lipid hydroperoxides

The attack of LOO^•^ on the adjacent polyunsaturated fatty acids yields C9-OOH and C13-OOH hydroperoxides with a concomitant formation of a conjugated dienes on the adjacent carbons. The short-lived lipid hydroperoxides decompose to a variety of the secondary product such as aldehyde, epoxide, keto or hydroxy compounds [47]. Among the aldehyde products, MDA is the secondary product formed by the oxidation of polyunsaturated fatty acid. Our results showed that the addition of linoleic acid to the cells results in the formation of MDA (Fig. 3C). The enhancement in the MDA formation in the disrupted cells is caused by enhanced formation of ROS or higher accessibility of the linoleic acid to the lipoxygenase in the disrupted cells.

### Triplet excited carbonyl and singlet oxygen formation

The attack of LOO^•^ on the adjacent polyunsaturated fatty acid results in the formation of another L^•^, which initiates the formation of another LOO^•^. When the concentration of LOO^•^ increased, the interaction of LOO^•^ with another LOO^•^ becomes feasible. Self-reaction of LOO^•^ by Russell-type of mechanism yields ^3^(C = O)^*^ and molecular oxygen or the ground state of carbonyls (C = O) and ^1^O_2_ (Fig. 6). It has been previously demonstrated that the formation of ^3^(C = O)^*^ and ^1^O_2_ proceeds via a tetraoxide intermediate [48]. The electronic transition of ^3^(C = O)^*^ to the singlet ground state is accompanied by the blue-green phosphorescence (λ_max_ at 450–550 nm), whereas the bimolecular reaction of ^1^O_2_ leads to the dimol red emission (λ_max_ at 634 and 703 nm).

### Excitation energy transfer from triplet excited carbonyls to chlorophylls

Our observation that the photon emission in the blue region of the spectrum is neglectable (Fig. 5A, trace c) shows that ^3^(C = O)^*^ does not contribute significantly to the ultra-weak photon emission. The findings that the photons are emitted predominantly in the red region of the spectrum might indicate that ^1^O_2_ is the main emitter of ultra-weak photon emission. However, our observation that ^1^O_2_ scavenger (histidine) has no effect on the ultra-weak photon emission reveals that ^1^O_2_ is unlikely involved in the ultra-weak photon emission (Fig. 5, trace b). The detail exploration of spectral properties in the red region of the spectrum showed that the maximum photon emission is at 680 nm which corresponds to the photon emission of chlorophyll molecules (Fig. 5C).

These considerations reveals that in the chlorophyll-containing sample, the excitation energy from ^3^(C = O)^*^ is transferred to chlorophyll molecule (Fig. 6). In the triplet-singlet energy transfer mechanism, the triplet excitation energy from carbonyls is transferred to chlorophyll molecule forming singlet excited state of chlorophyll (^1^Chl^*^) [49], [50]. In the triplet-triplet energy transfer mechanism, the triplet excitation energy from carbonyls is transferred to the chlorophyll molecule forming the triplet excited state of chlorophyll (^3^Chl^*^) which is converted to ^1^Chl^*^ by reverse intersystem crossing [51]. In the agreement with our observation, it has been previously demonstrated that the exposure of thylakoid membranes to heat stress is accompanied by the photon emission from chlorophyll molecules [21], [27], [38], [52]. More recently, Kobayashi and co-authors (2007) demonstrated that a chlorophyll molecule serves as the final emitter of photons during hypersensitive response to cucumber mosaic virus in cowpea.

### Conclusion

The present study provides the evidence on the involvement of lipid peroxidation in the ultra-weak photon emission measured in *Chlamydomonas reinhardtii* cells. It is proposed that the ultra-weak photon emission can be a useful non-destructive tool to follow the extent of lipid peroxidation in the photosynthetic organism under *in vivo* conditions. Detection of ultra-weak photon emission, which provides both temporal and spatial information on the lipid peroxidation opens new possibilities to better characterize the response of cyanobacteria, algae and plants to various stresses. The direct detection of ultra-weak photon emission using CCD camera, which provides an information on the spatial distribution of the photon emission in the sample, characterizes the different responses of plant to the stress factors in the different parts of the leaves. Highly sensitive PMT, which provides an information on the kinetics of ultra-weak photon emission, enables to follow the temporal characteristics of the plant response to the environmental stress factors. The use of ultra-weak photon emission as a non-invasive diagnostic tool for monitoring of lipid peroxidation helps to better understand the mechanistic insights into the plant response to the numerous abiotic and biotic stresses.

## Materials and Methods

### *Clamydomonas reinhardtii* grown conditions

*Chlamydomonas reinhardtii* algae strain (wild type: CC-002) was obtained from the Chlamydomonas Genetic Center (Duke University, Durham, NC, USA). The cells were grown in a continuous white light (100 µmol m^−2^ s^−1^) in Tris-Acetate-Phosphate (TAP) medium in which acetate represents the main carbon source. The algal culture was placed on a multi-position magnetic stirrer RT 5 power (IKA Werke GmbH, Staufen, Germany) and permanently stirred to obtain constant CO_2_ concentration in the growing medium. The cells were studied during the stationary growth phase at a density of approximately 7×10^7^ cells ml^−1^. Cell density was determined by a manual microscopic cell count.

### Ultra-weak photon emission measurement

*Chlamydomonas reinhardtii* cells in TAP medium (total volume of 2 ml) placed on the glass Petri dish (3 cm diameter) were put in the front of CCD camera or PMT window. For two-dimensional photon emission imaging, the photons were reflected by the mirror to the vertically situated window of the CCD camera. For one-dimensional photon counting, Petri dish was placed at a distance of 3 cm below the horizontally situated PMT window. To eliminate the interference by delayed luminescence, the cells were dark-adapted for 30 min prior to the measurements. During the measurements, 2 mM linoleic acid was added to the cells and mixed thoroughly. In some measurements, 200 mM mannitol, 5 mM catechol and 10 mM histidine were added to the cells prior to the measurements. Molecular oxygen was removed by glucose/glucose oxidase enzymatic system by the addition of 1 mM glucose in 50 U ml^−1^ glucose oxidase (EC 1.1.3.4, from *Aspergillus niger*) (Fluka). During the addition of oxygen trap, the air was removed from the upper part of the cuvette by a gentle stream of nitrogen gas and the cuvette was sealed with the help of a 1 cm thick rubber cap and paraffin wax. The presented traces are representative of data which is measured at least 3 times.

### Highly sensitive CCD camera and photon counting

Highly sensitive CCD camera VersArray 1300B (Princeton instruments, Trenton, NJ, USA) was used for two-dimensional photon imaging. To reduce the dark current, CCD camera was cooled down to −110°C using a liquid-nitrogen cooling system. The CCD camera was equipped with a 50-mm focal distance lens with an f-number of 1.2 (F mount Nikkor 50-mm, f:1.2, Nikon) to enhance the light collecting efficiency. Spectral sensitivity of CCD camera was within the range of 200–1000 nm with the almost 90% quantum efficiency in the visible range of the spectrum. The spectral sensitivity was limited to 350–1000 nm by the lenses. The data correction was made by subtracting the background noise before every measurement. The measurement was done in the image format of 1340×1300 pixels. CCD camera parameters were as follows: scan rate, 100 kHz; gain, 3; accumulation time, 30 min.

One-dimensional photon counting was measured using low-noise photon counting unit C9744 (Hamamatsu Photonics K.K., Iwata city, Japan). To reduce the thermal electrons, PMT was cooled down to −30°C using thermoelectric cooler C9143 (Hamamatsu Photonics, K.K., Iwata city, Japan). The PMT was kept vertically to minimize the dark counts to approximately 2 counts s^−1^ at −1150 mV. The spectral sensitivity of the PMT was within the range of 160–710 nm.

The CCD camera and PMT were situated in the experimental dark room with a dimension 3 m×1.5 m×2.5 m. The whole interior of the experimental dark room was painted with black color. The door in the experimental dark room was protected completely with a black curtain to restrict any external light. The data recording computer was installed in the operation dark room.

For the spectral analysis of ultra-weak photon emission in the blue and the red region of the spectrum, the bandpass filter BG 14 (310–650 nm) (Schott & Gen., Jena, Germany) and the edge filter RG 6 (spectral range >600 nm) (Schott & Gen., Jena, Germany) were used. Further, the spectral analysis of photon emission in the red region of the spectrum was explored with a set of interference filters with a bandwidth of 10 nm centered at 641 nm, 651 nm, 662 nm, 671 nm, 683 nm, 691 nm and 702 nm (Andover Corporation, Salem, USA).

### Determination of Thiobarbituric acid reactive substance (TBARS)

The extent of lipid peroxidation under the exogenous application of linoleic acid was estimated by measuring the formation of thiobarbituric acid reactive substance (TBARS) as described in Halliwell and Chirico [18] with minor modifications. After addition of linoleic acid to the cell culture (3 ml), butylated hydroxytoluene at a final concentration of 0.01% (w/v) was used to terminate the lipid peroxidation chain reaction. The cells were harvested by centrifugation at 5000× g. The pellet was mixed with 2 ml of 80 mM TBA and heated in a boiling water bath for 10 min. After cooling down to 25°C, the reaction mixture was centrifuged at 8500× g for 5 min to obtain a clear supernatant. Thiobarbituric acid reactive substance was determined by absorbance at 532 nm using Spectrophotometer Unicam UV 550 (ThermoSpectronic, Cambridge, UK). The amount of (TBA)_2_-MDA adduct was determined using of a molar extinction coefficient 1.54×10^5^ M^−1^ cm^−1^ [11]. The presented data are expressed as mean and standard error of the mean of at least three measurements (mean±S.E.M, *n = 3*).
